# Fabrication of Cost-Effective Microchip-Based Device Using Sandblasting Technique for Real-Time Multiplex PCR Detection

**DOI:** 10.3390/mi15080944

**Published:** 2024-07-24

**Authors:** Yiteng Liu, Zhiyang Hu, Siyu Yang, Na Xu, Qi Song, Yibo Gao, Weijia Wen

**Affiliations:** 1 Division of Emerging Interdisciplinary Areas, Academy of Interdisciplinary Studies, The Hong Kong University of Science and Technology, Clear Water Bay, Kowloon, Hong Kong SAR 999077, China; yliudx@connect.ust.hk (Y.L.);; 2 Thrust of Advanced Materials, The Hong Kong University of Science and Technology (Guangzhou), Nansha, Guangzhou 511400, China; 3 Department of Physics, The Hong Kong University of Science and Technology, Clear Water Bay, Kowloon, Hong Kong SAR 999077, China; 4 Shenzhen Shineway Technology Corporation, Shenzhen 518048, China

**Keywords:** multiplex polymerase chain reaction (mPCR), sandblasting, microchip fabrication, microdevice

## Abstract

The combination of multiplex polymerase chain reaction (mPCR) and microfluidic technologies demonstrates great significance in biomedical applications. However, current microfluidics-based molecular diagnostics face challenges in multi-target detection due to their limited fluorescence channels, complicated fabrication process, and high cost. In this research, we proposed a cost-effective sandblasting method for manufacturing silicon microchips and a chip-based microdevice for field mPCR detection. The atomic force microscopy (AFM) images showed a rough surface of the sandblasted microchips, leading to poor biocompatibility. To relieve the inhibitory effect, we dip-coated a layer of bovine serum albumin (BSA) on the irregular substrate. The optimized coating condition was determined by scanning electron microscope (SEM) and energy-dispersive X-ray spectroscopy (EDS) (65 °C for 60 min). After sufficient coating, we performed on-chip PCR tests with 500 copies/mL Coronavirus Disease 2019 (COVID-19) standard sample within 20 min, and the sandblasted microchip displayed a higher amplification rate compared to dry etching chips. Finally, we achieved a 50 min mPCR for screening five resistance genes of the endophthalmitis pathogens on our microdevices, with strong specificity and reliability. Thus, this sandblasted microchip-based platform not only provides a rapid, accessible, and effective solution for multiplex molecular detection but also enables large-scale microfabrication in a low-cost and convenient way.

## 1. Introduction

Since the twenty-first century, constantly emerging infectious diseases such as severe acute respiratory syndrome (SARS), Ebola virus disease (EVD), and Coronavirus Disease 2019 (COVID-19) have seriously endangered human health and social stability [1,2,3]. Nucleic acid amplification testing (NAAT) allows early screening and identification of pathogenic microbes, which is of crucial importance to either timely pandemic control or satisfactory patient outcomes [4]. Regarded as a “gold standard” of NAAT-based molecular diagnoses, real-time polymerase chain reaction (PCR) techniques are widely used in clinical practice due to their robust sensitivity and specificity [5]. Multiplex PCR (mPCR) refers to conducting amplification and detection of various genetic materials in the same reaction involving a set of different primers labeled by fluorescent dyes [6]. Multiplexing detection shows the advantages of saving raw reaction materials and minimizing repeated pipetting compared to separate assays, enabling it to become a preferred alternative in screening various pathogens with similar symptoms and drug-resistance genes [7,8]. For example, multiple PCR assays can assist physicians in determining antibiotic tolerance of pathogenic bacteria, making medication guidance safe and effective. However, tedious sample-to-answer time, cumbersome dimensions, and dependence on professional laboratory settings are the common drawbacks of conventional PCR-based methods [9]. These weaknesses would obstruct its further application in the healthcare field, especially for resource-limited regions. In consequence, aiming to realize multiplex molecular point-of-care testing (POCT), a rapid, field-deployable, cost-saving, and flexible detection strategy is a pressing demand.

A microfluidic chip, also called a lab-on-a-chip, is defined as manipulating and processing a small volume of fluid at a microscale, which can systematically integrate multiple biochemical steps into one tiny unit via microchannels, microchambers, micropumps, microvalves, and so on [10,11]. Microchip-based devices benefit bioanalyses such as NAATs, with efficient thermal transmission, low reagent consumption, miniaturized instrument size, and a high level of integration, satisfying the requirements of POCT scenarios [12,13]. Over the years, global COVID-19 prevalence stimulated the development of biomedical diagnosis, resulting in many advanced and powerful microfluidic devices that are potentially suitable for on-site pathogen detection [14,15]. For instance, Oliveira et al. reported a polystyrene-toner based microfluidic chip and a centrifugal liquid mixing method, which can generate a visual result of COVID-19 detection through a 10 min isothermal amplification [16]. Cojocaru et al. evaluated a commercial product named AriaDNA analyzer using a microchip that spatially separated three microwells to detect two target genes of COVID-19 and an internal reference gene [17]. M.G. Rizz et al. utilized a 6-microwell biochip equipped with a portable heating device to identify various bacterial pathogens [18]. Nevertheless, when it comes to multiple target detection, these diagnosis schemes show insufficient performance due to their limited sample capacity and fluorescence channels. Some of these methods improve the target numbers of the single test by means of developing more reaction chambers on the chip, leading to a restricted sample amount per run and repetitive pipetting operation. Thereby, a miniaturized device with multiple fluorescence channels and an adequate sample capacity can tackle these issues while ensuring high detection efficiency.

Regarding cost control, the fabrication process of the microchip should be considered as an important issue, since it regularly uses as disposable consumables. Typically, microfabrication includes chip design, substrate preparation, pattern transfer, etching, surface modification, bonding, and dicing [19]. Among them, pattern formation and etching are generally the dominant part of the total cost. Photolithography and wet and dry etching technologies are widely utilized for developing planar and three-dimensional structures on the silicon substrate [20,21]. However, these methods require complex protocols, high-cost instruments, professional training, and handling toxic and environmentally harmful chemicals [22]. Hot embossing could generate microstructures in a few steps, which is feasible for mass production, but it is not usually used on the silicon-based substrate, and mold fabrication is tedious and expensive at the proofing stage [23]. Using a precise laser beam, the laser ablation technique is used to pattern silicon wafers within a short running time; however, it requires sophisticated instruments [24,25]. To overcome these challenges, many efforts have been made to build a simple, inexpensive, and convenient solution to fabricate microfluidic components for prototyping demos or mass production, especially in resource-limited conditions [26,27,28]. However, up to now, there is little research on sandblasted silicon microchips in biological applications. The sandblasting technique is applied to physically etch texture on the material surface using high-speed abrasives [29]. Eun et al. present a micro-sandblaster etching process on the silicon wafer with a dry photoresist and demonstrated that both sandblasting and wet etching could develop microchannels with ideal geometric characterization for bioanalytical purposes; however, they did not show substantial evidence of biochemical reaction on the sandblasted microchip [30]. Although this technique shows lower precision compared with plasma etching or deep reactive ion etching (DRIE), it can develop specific microstructures in a simpler, more accessible, and faster way [31]. With these advantages, sandblasting would likely become a feasible strategy for mass production in the microfluidic industry, and further study is urgently needed.

To practically implement on-site mPCR detection, the fabrication cost of the microchip should be reduced to an affordable level, while its matched microdevice must be field-portable, reliable, and offer multichannel and rapid operation. Our earlier work proposed a silicon-based microheater and microfluidic chips that were fabricated by photolithography and DRIE, which can effectively minimize the instrument size and speed up reaction time [32,33]. These methods show less feasibility for upscaled applications, because their fabrication processes demand costly facilities and lengthy procedures. In this research, we designed a low-cost manufacturing process for microfluidic chips using the sandblasting technique and a wash-free mask. Meanwhile, we assembled a silicon heater-based microdevice with five motor-driven fluorescence channels, enabling rapid thermal cycling and multiple target detection. The dimension of the device is 380 mm × 245 mm × 245 mm, with a weight of 6.8 kg, which is easy to deploy for field detection. The microdevice allows on-chip amplification and fluorescence analysis for six samples in a single run. It is noted that the blasted microchamber surface is much rougher than that of DRIE, likely resulting in poor biocompatibility [34,35]. Published studies proposed physical polishing [36] and chemical etchants [37] for reducing the roughness of silicon surfaces, though these may cause a rise in workload and production cost. For solving this problem, bovine serum albumin (BSA) is chosen to improve the biological compatibility of the sandblasted surface by relieving the absorption effect between the wafer surfaces and DNA polymerase, thereby facilitating the reaction [38]. To obtain a satisfactory protein coating, we optimized the immersion time and incubation temperature by approximately measuring the concentration of the absorbed BSA. After the BSA coating, the sandblasted microchip can achieve PCR assays whose amplification efficiency is similar to the microfluidic chips etched by DRIE. Finally, we successfully performed multiple on-chip PCR for screening the drug resistance of genes of the endophthalmitis bacteria with high specificity and robustness. Hence, through a low-cost, improved, and productive fabrication process, we think the point-of-care microdevice with sandblasted microchips will become a promising mPCR strategy for primary medical care.

## 2. Materials and Methods

### 2.1. Design of the Portable Microdevice

**Figure 1:**
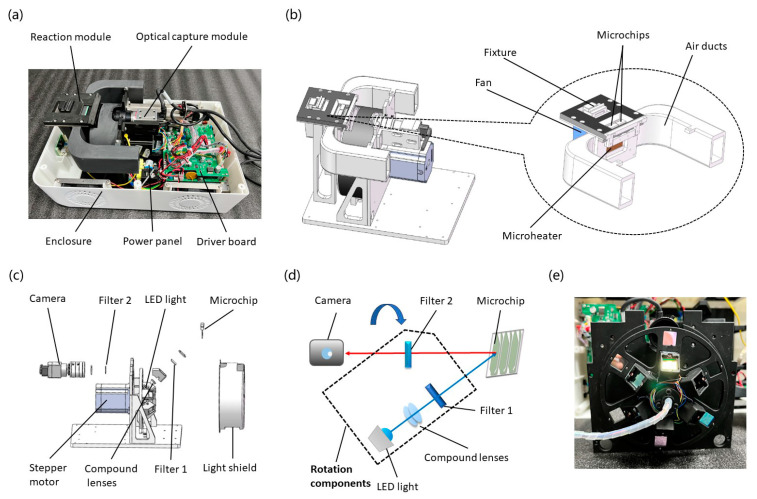
The photos and design scheme of the microdevice. (**a**) Internal structure image of the prototype with a bottom case (acrylonitrile-butadiene-styrene (ABS)). (**b**) The schematic of the reaction module. (**c**) The schematic of the optical capture module. (**d**) The principle of the fluorescence collection. (**e**) The photo of the rotation components.

The microdevice mainly consists of a reaction module, an optical capture module, a customized driver board (Xingbiao ELECTRONIC, Shenzhen, China), a power panel (LOF120, MORNSUN, Guangzhou, China), and a 3D-printed enclosure (Figure 1a). More specifically, temperature variation was achieved by a silicon microheater, as presented in our previous study [33], and a tiny fan (9GAX, Sanyo Denki, Tokyo, Japan). The microheater integrated heating and temperature sensing functions, and a proportional integral derivative (PID) control ensured the temperature accuracy. As shown in Figure 1b, microchips (silicon side) were tightly pressed against the microheater by a fixture, which formed a sandwich structure where nucleic acid was amplified. Moreover, a pair of air ducts was applied to increase cooling efficiency. On the other hand, as seen in Figure 1c, we designed a miniaturized optical path involving a CMOS camera (a2A1920, Basler, Ahrensburg, Germany), a camera lens (M0814-MP2, Computar, Mebane, NC, USA), a set of filters (excitation filters: EX470/30, EX530/20, EX570/20, EX630/20, and EX675/20; emission filters: EM520/20, EM565/22, EM612/20, EM675/20, and EM725/20, NANOMACRO, Shenzhen, China), compound lenses (KEDING OPTICAL TECHNOLOGY INC., Hangzhou, China), and LED lights (G45P470KN, G45P530KN, E42N580KN, E42N630KN, and G42N670KN, Yingfeng Optoelectronics, Shenzhen, China), which is available for five fluorescence channels (FAM, HEX, ROX, Cy5, and Cy5.5). As described in Figure 1d, a battery of lenses was used to maintain parallel and uniform LED light. Then, the excitation light (blue arrow) was propagated through filter 1 and excited the sample. The emission light (red arrow) was obtained from the excited probes and eventually reflected to the CMOS camera after passing filter 2. To enhance the utilization rate, a stepper motor (57CM, Leadshine, Shanghai, China) was used to switch optical components for different light collections. During the reaction and fluorescence collection process, all modules were controlled by a driven board using customized computer software (V1.0).

The Ct value of the reaction was the cycle number where the fluorescence curve intersects the threshold. We calculated the threshold by a multiple of the standard deviation based on the fluorescence intensity of the initial cycles [39]. Moreover, the sigmoid function was used to conduct curve-fitting during the data analysis process [40].

### 2.2. Fabrication of the Glass-Silicon Microfluidic Chip

**Figure 2:**
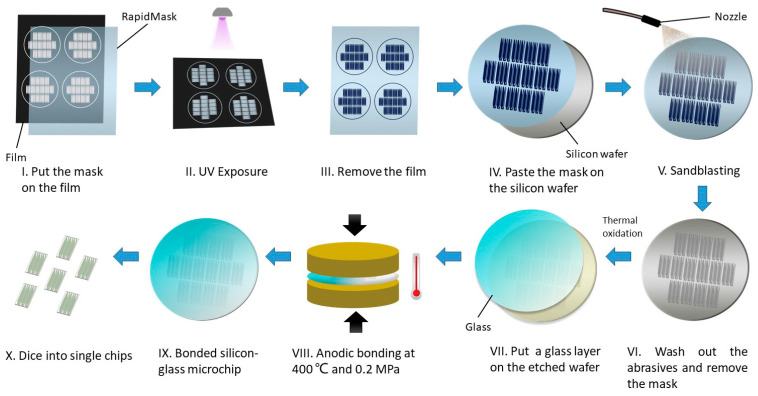
Illustration of 3-well glass-silicon microchip fabrication process using sandblasting technique and RapidMask to develop patterns on the silicon substrate.

The workflow of the sandblasted microchip is depicted in Figure 2. The 4-inch silicon wafers and 4-inch glass wafers were provided by the Nanosystem Fabrication Facility (CWB) of the Hong Kong University of Science and Technology (Hong Kong, China). Here are the steps we followed: (I) Firstly, the commercial RapidMask purchased from IKONICS Imaging (SCCF446II25302, Duluth, MN, USA) was stacked on the patterned film printed by a laser printer. The transparent part of the film represents the blasting area. (II) After 2 min UV exposure, the photoresist covered by the clear film became fragile, while the rest remained rigid. (III) We removed the film and cut the mask into a size to fit with the 4-inch silicon wafer. (IV) The adhesive side of the mask was pasted on the silicon substrate, and bubbles between the mask and substrate were released with a wheel brush. (V) The sandblasting was conducted in the automated sandblaster (PF2400, Comco Inc., Burbank, CA, USA), connected with a 0.6 MPa compressed air source. We employed aluminum oxide as abrasive and blasted the silicon at a working pressure of 0.3 MPa. The blasting distance between the moving nozzles and silicon was 4 inches. The total processing time was approximately 20 min. (VI) We washed out the residual abrasive with deionized (DI) water and tore off the mask. (VII) The silicon substrate then developed an oxide layer by thermal oxidation. (VIII and IX) Then, utilizing custom-made hot-pressing equipment, the patterned silicon wafer was bonded with a glass wafer by anodic bonding. (X) Lastly, the bonded wafer was diced into single 3-well microchips (12 mm × 23.3 mm) and simultaneously formed inlets and outlets on top of the chip. Another microchip mentioned in our early study was prepared by standard photolithography and DRIE.

### 2.3. Temperature Features of the Microheater-Based Reaction Module

The microheater was designed to provide two microchips with certain temperature conditions. We measured the heating and cooling rate and temperature uniformity of the heater via a data logger (34970A, Keysight, Santa Rosa, CA, USA). To obtain the real temperature of different microwells, a 0.5 mm silicon slice with four K-type sensors was used to detect the temperature curve. We set three temperature cycles between 45 °C and 95 °C, with a 30 s duration time and a record frequency of 5 times/s. The average heating and cooling rates were calculated by the ratio of temperature variation and consuming time. Similarly, we set thermal cycles of 55 °C, 75 °C, and 95 °C, with 90 s duration time, for 1 cycle, and recorded the temperature values 1 time per second. During the isothermal stage, the difference between maximum and minimum values was regarded as the temperature uniformity.

### 2.4. Fluorescence Reliability of the Optical Module

Fluorescent dyes used in the repeatability test include fluorescein sodium salt (CAS No.: 518-47-8), atto 532 (CAS No.: 06699, Sigma-Aldrich, Inc., St. Louis, MO, USA), 6-ROX Acid (CAS No.:194785-18-7), Sulfo-Cyanine 5 (CAS No.:146368-11-8), and Sulfo-Cyanine 5.5 (CAS No.:210892-23-2, MedChemExpress LLC, Monmouth Junction, NJ, USA) for FAM, HEX, ROX, Cy5, and Cy5.5 channels, respectively. Each fluorescent dye was diluted to three kinds of concentrations (FAM: 1.8, 0.45, and 0.1125 μM; HEX: 1, 0.25, and 0.0625 μM; ROX: 2, 0.5, and 0.125 μM; Cy5: 0.4, 0.1, and 0.025 μM; Cy5.5: 2, 0.5, and 0.125 μM). The optical module performed signal collection 15 times for each concentration. Then, we performed single-target amplification tests on the microwells simultaneously to determine fluorescent overlap interference. The calibration kits and positive references were provided by Shineway Technology (Shenzhen, China). The PCR program was conducted under the following conditions. (1) 95 °C for 180 s, 1 cycle; (2) 95 °C for 10 s and 60 °C for 40 s, 40 cycles. To verify the roughness effect on the fluorescence collection, we replicated signal capture 20 times using sandblasted and DRIE microchips that were loaded with low and high concentrations of FAM fluorescein.

### 2.5. Process Optimization of the BSA Dip-Coating and Surface Characterization of the Microchips

To improve the biocompatibility of the sandblasted microchip, we used a recommended concentration (5 mg/mL) of BSA (CAS No.: 9048-46-8, reagent grade, Sigma-Aldrich, Inc., St. Louis, MO, USA) to passivate the rough surface [16]. After thermal oxidation, the carved silicon wafer was diced into small pieces and then dipped in the EP tube with 1 mL 5 mg/mL BSA solution. We incubated the tubes in the water bath at 37 °C for 10 min, 30 min, 60 min, and 120 min. As for the incubation temperature, the BSA coating was treated at 25 °C (room temperature), 37 °C, and 65 °C for the optimized processing time. All coated silicon pieces were dried in the electrothermal drier for follow-up analysis. Atomic force microscopy (AFM) (Dimension icon, Bruker, Karlsruhe, Germany) was applied to characterize the surface morphology of silicon microwells developed by different techniques. The samples were scanned with a 20 μM × 20 μM scale, using a Scan-Assyst probe in the air environment. The roughness was calculated with Nanoscope Analysis (version 2.0, Bruker, Karlsruhe, Germany). The coating effects of different treatments were observed by scanning electron microscope (SEM) (JSM-7100, JEOL (Europe), Nieuw-Vennep, The Netherlands). The morphological properties of DRIE microchips were used for comparison. Meanwhile, we utilized energy-dispersive X-ray spectroscopy (EDS) to measure the atomic percentage of nitrogen, carbon, oxygen, and silicon. The amount of absorbed BSA was estimated based on the proportion of nitrogen, since the N element only derives from BSA in this experiment. Finally, the optimization condition was applied to handle the diced microchips in the vacuum environment.

Besides this, we added the BSA-coated silicon piece in the EP tube with BSA solution and then performed thermal circulation in the PCR analyzer (FQD-96A, Hangzhou Bioer Technology Co., Ltd., Hangzhou, China), aiming to validate the stability of the coating layer in practical application (Figure S3a). After a standard 45-cycle reaction, the aged piece was dried of remaining water for further AFM surface characterization. The PCR program is detailed in Section 2.6.

### 2.6. COVID-19 PCR Detection Performed on the DRIE and Sandblasted Microchips

In this research, we performed on-chip PCR assays in the microdevice using a Novel Coronavirus (SARS-CoV-2) Fast Nucleic Acid Detection Kit (lyophilized powder) offered by Cowin Biotech (Taizhou, China). The RNA standard sample of the Omicron variant containing the ORF1ab gene and N gene was bought from the China National Institute of Metrology (Beijing, China). We diluted the template to 500 (detection limit concentration) and 2000 copies/mL and added them to the redissolved PCR solution according to the user manual. Afterward, a 12 μL mixture was pipetted to the microchambers under the capillary effect, and the microchips were closed by a plug-to-run sealer, preventing evaporation during thermocycling (Figure S1). The reaction program was set up as follows: (1) Reverse transcription: 55 °C for 1 min, 1 cycle; (2) Pre-denaturation: 95 °C for 20 s, 1 cycle; (3) Amplification: 96 °C for 5 s and 58 °C for 10 s, 45 cycles. The ORF1ab gene and N gene were detected by FAM and HEX channels, respectively. The same protocol was carried out in the DRIE microchip. With the equal sample concentration, we compared the Ct value difference to assess the biocompatibility between the two fabrication methods.

### 2.7. Multiplex PCR Assay Protocol

With improved biocompatibility, we tested multiple drug-resistant genes (MecA, AmpC, OXA23, VIM, and SHV-1) of the endophthalmitis pathogen on the sandblasted microchips with our microdevice. The PCR reagent kit with positive references was purchased from Shineway Technology (Shenzhen, China). Single genes and mixed target genes were used to validate the specificity of the microfluidic mPCR. We built a 25 μL reaction mixture with a 5 μL template, following the manual, and 12 μL of was loaded into the chip. The sample loading and sealing processes were the same as detailed in the previous section. The thermal cycle was applied in two steps. (1) 95 °C for 3 min, 1 cycle; (2) 95 °C for 10 s and 60 °C for 40 s, 45 cycles; five fluorescence channels were activated for signal collection. DI water was used as a negative control in this research.

## 3. Results and Discussion

### 3.1. Thermal Cycle Performance

**Figure 3:**
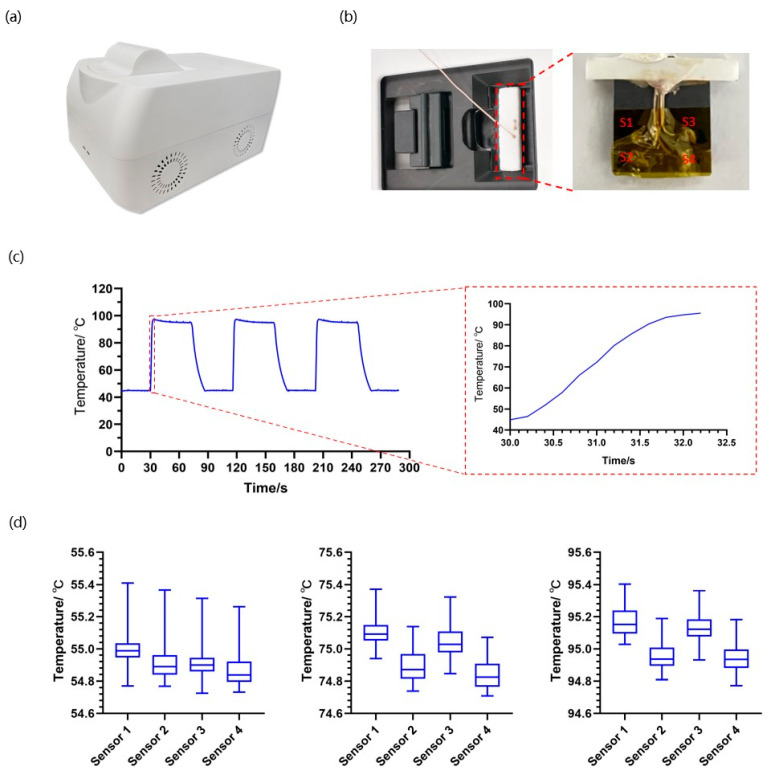
(**a**) Appearance of the microdevice (380 mm × 245 mm × 245 mm). (**b**) The detector is inserted into the reaction module for temperature recording (top view of the microdevice and the distribution of four temperature sensors. (**c**) Temperature curve of the microheater (thermal cycles from 45 °C to 95 °C). (**d**) Temperature uniformity of the microheater at 55 °C, 75 °C, and 95 °C.

Temperature properties play a decisive role in real-time PCR, affecting its amplification efficiency, reliability, and even success [41]. Based on the joule heating, the microheater was fabricated by sputtering patterned platinum on the silicon substrate, forming thin-film resistances for rising and sensing temperatures. With the integrated microfluidic heater, we miniaturized the detection system to a compact size, as showcased in Figure 3a. We applied a silicon sheet with the same thickness as the microchip to monitor the thermal profile of the microheater. As seen in Figure 3b, the sheet with 4 K-type sensors was connected tightly to the microheater during the thermal circulation, leading to more precise measuring of the practical temperature of the microchamber. Generally, the average heating rate is more reflective of thermal variation performance than the instantaneous rate. Under the PID control, the temperature ramped up dramatically at the initial stage, and the heating speed decreased gradually as it approached the target temperature (Figure 3c). The profile showed that the temperature rises rapidly, with an average heating rate of 22.73 °C/s, which was much higher than commercial rapid PCR devices, which range from 4 °C/s to 10 °C/s [42]. In addition, the cooling rate was approximately 3.73 °C/s, using an axial fan and air ducts. Compared with other materials, the high heat conductivity of silicon material contributes to effective thermal cycling [43]. Though the cooling rate of the microdevice is comparable with other instruments, the high heat conductivity of silicon material contributes to effective thermal circulation. For example, the running time of a 45-cycle COVID-19 assay can be notably shortened to less than 20 min on our platform. The duration of the isothermal phase is consistent with the kit manual, ensuring specific amplification. As shown in Figure 3d, the silicon-based heater demonstrated homogenous thermal distribution, with maximum differences of 0.685 °C, 0.662 °C, and 0.630 °C at 55 °C, 75 °C, and 95 °C, respectively. All of the measured values fell within a tolerance of ±0.5 °C from the target temperatures, showing accurate thermal control. We also found sensor 2 and 4 recorded slightly lower temperatures than those tested by others, which may result from wiped joints at the backside of the microheater.

### 3.2. Fluorescence Collection Performance

**Figure 4:**
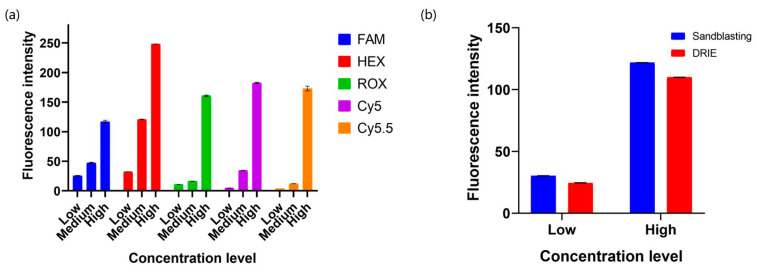
(**a**) Fluorescence repeatability of the optical module using low, medium, and high concentrations of fluorescein. (**b**) Comparison of the fluorescence intensity between sandblasted and DRIE microchips with low and high levels of FAM fluorescein. Error bar represents the deviation of repeated tests.

As illustrated in Figure 4a, average fluorescence intensity exhibited an upward tendency as the dye concentration increased in all channels. Fluorescence channels showed various intensity increments due to different capture sensitivities toward excitation lights. All concentrations displayed minor deviations, and their coefficients of variation (CV) were lower than 3% (Table S1), indicating strongly reproducible results. Moreover, there was no signal crosstalk between different fluorescence channels (Figure S2). Figure 4b shows that slightly stronger signals were collected from the sandblasted surface compared to DRIE chips, and both kinds of microchips can obtain repeatable results. Thus, the roughness difference indicated no noticeable effects on fluorescence reading.

In comparison to traditional multi-fluorescence systems, a notable strength of our optical module is the miniaturization design. As summarized in Table 1, the size of our microdevice is only about a third of the size of desktop PCR instruments like the ABI 7500 fast (Thermo Fisher Scientific, Waltham, MA, USA) [44] and LightCycler^®^ 2.0 System (Roche Diagnostics, Rotkreuz, Switzerland) [45]. To bring about this outcome, we applied mini filters and lenses to condense the optical module volume. Transparent and planar properties of the glass-silicon chips also made it possible to simplify the optical path (Figure 1d). From another perspective, we enhanced the camera utilization rate and further lowered costs by rotating LEDs and filters toward multiple channels. Contrary to commercial benchtop PCR instruments (~23,000~55,000 USD) [46], the production cost of the sandblasted microdevice decreased to an acceptable level (USD ~6500) due to the miniature optical and microheater-based reaction modules. With characteristics of transportable dimensions, cost effectiveness, and flexibility, this optical module can provide microfluidic platforms with reliable fluorescence signal collection.

### 3.3. AFM Scanning and Roughness Analysis of the Microchips

**Figure 5:**
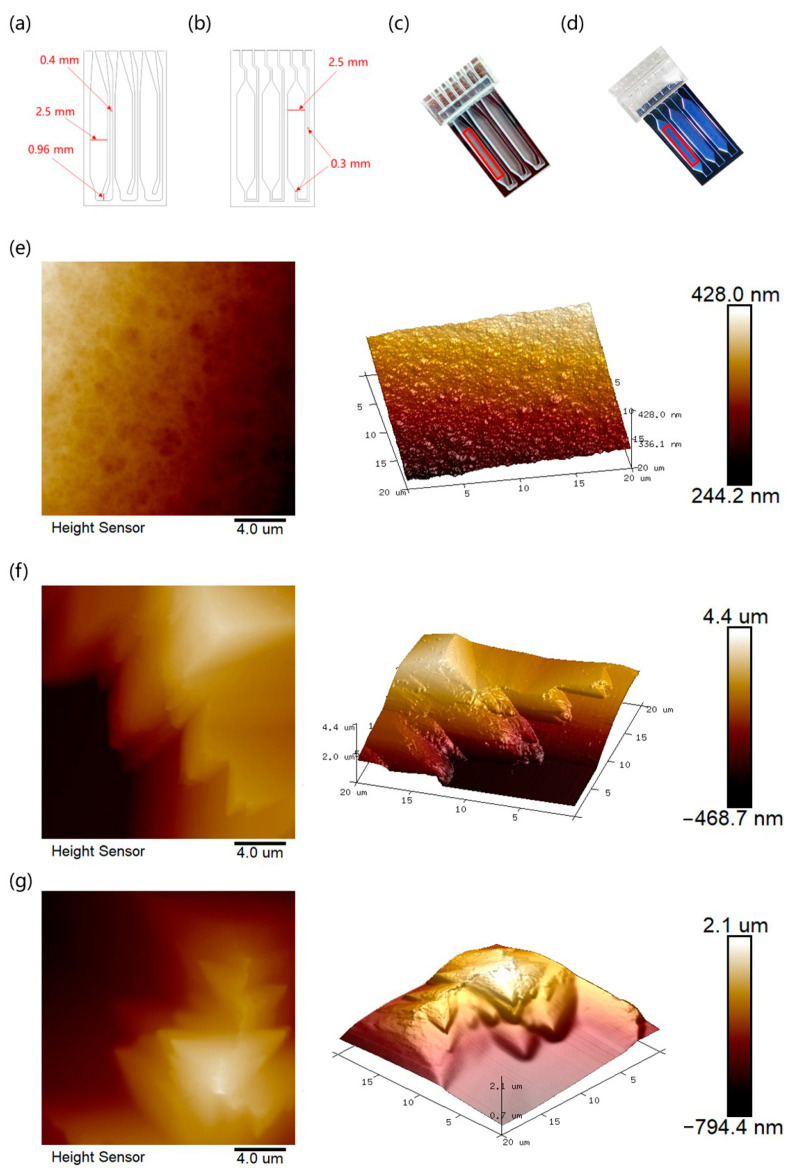
Design schematic of the (**a**) sandblasted and (**b**) DRIE microchannels. Photos of (**c**) sandblasted and (**d**) DRIE microchips (AFM analyzed the marked areas of microwells). The 20 μm × 20 μm AFM surface morphology of silicon substrate processed by (**e**) DRIE, (**f**) sandblasting without BSA coating, and (**g**) sandblasting with BSA coating (60 min dipping time at 37 °C).

To obtain a cost-effective and easy-to-operate etching process, we used a hard film named RapidMask to transfer the patterns. As shown in Figure 5a,b, the sandblasted and DRIE microchannels were designed with a width scope of 0.4 mm to 0.96 mm and 0.3 mm, respectively. The key factor driving this design is the fabrication precision difference between the two methods. Generally, dry etching can achieve nanoscale processing that is widely used in electronic chip manufacture [47]. In this case, though microstructures developed by the sandblasting technique demonstrated lower dimensional accuracy than DRIE, hundreds of microns can fulfill the major demands of microfluidic manipulation for biomedical applications. After sandblasting, 300 μm deep microwells were mutually independent, to avoid cross-contamination. Finer structures can be visually observed from microchips etched by DRIE in contrast with sandblasting, as shown in Figure 5c,d. 

In this case, AFM was applied to quantify the roughness between the two techniques.

From the microscopic images described in Figure 5e,f, we can see that the silicon surface handled by sandblasting is much rougher than produced by DRIE. There were abundant sharp corners and edges after abrasive carving. As indicated in Table 2, the average roughness (Ra) and root mean square roughness (Rq) of the DRIE microchip were 2.41 nm and 3.04 nm, respectively. On the contrary, the sandblasted microchip displays a micron scale with an Ra of 344 nm and Rq of 416 nm, which was over a hundred-fold that of DRIE. Interestingly, we found that the sandblasted microchip roughness was reduced to 196 nm and 256 nm after BSA coating. However, the maximum roughness values of all sandblasting microchips were far greater than the DRIE chip, revealing that the BSA layer is essentially unable to change the rough property of the sandblasted surface. Research has revealed that a rough surface with large surface-to-volume ratio may result in inhibitory effects on biological reactions, because uneven features can absorb reactants and enzymes in the liquid environment [48]. Typically, buffer additives such as betaine, Tween, BSA, PEG (Polyethylene Glycol), and Dimethyl sulfoxide (DMSO) are used to improve the PCR process [38,49,50,51]. In this case, we aimed to relieve nonspecific adsorption of the sandblasted surface and facilitate reaction efficiency by a dip-coating method, thereby selecting protein BSA as a surface-modifying agent [52]. Figure 5g shows that the silicon surface becomes passivated after BSA immersion. Although BSA coating cannot significantly change the roughness of the surface to a smooth state, it can theoretically enhance material biocompatibility and lower the absorption effect. Furthermore, the BSA-coated sandblasted surface and edges were still passivated through 45 amplification cycles, indicating that the BSA layer has reliable stability (Figure S3b). Roughness characteristics of the aged surface are similar to initial values (Table 2).

Compared with the DRIE technique, the mask of sandblasting is achieved at a much lower price (1/11 of the DRIE mask), with a more flexible design, by film printing (Table 3). Sandblasters and abrasives provide more accessibility than dry etchers involving toxic fluorine-containing gasses. Consequently, the total cost of the sandblasted microchip is about half that of the DRIE microchip. In another respect, for massive-scale manufacturing, the sandblasting process can increase production efficiency by improving automatic control and the number of nozzles. Nevertheless, the DRIE process shows limited feasibility in batch fabrication due to its high equipment maintenance fee.

### 3.4. BSA Coating Optimization and SEM Analysis

**Figure 6:**
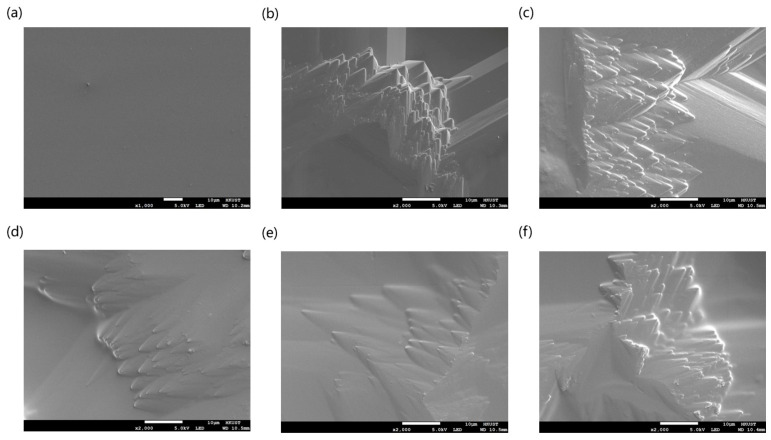
SEM images of silicon surface fabricated by (**a**) DRIE, (**b**) sandblasting without BSA coating, and sandblasting with (**c**) 10 min, (**d**) 30 min, (**e**) 60 min, and (**f**) 120 min BSA dipping at 37 °C.

A homogeneous layer with an adequate content of BSA is beneficial to reduce the absorption effect derived from rough surfaces. Generally, dip-coating conditions such as solution concentration, duration time, and incubation temperature significantly impact the protein layer development. In order to access and optimize the coating quality, we utilized SEM to characterize the major morphology of the BSA-coated surface. As shown in Figure 6, a more irregular plane was found on the sandblasted microchip with respect to DRIE-handled chips, which was similar to the AFM photos. As can be seen from the scanning images, the coating effect became distinct after 30 min of dipping, and sufficient BSA layers were obtained. Despite the existing stratified structure causing obvious height discrepancy on the silicon surface, the well-coated BSA layer made the sharp edges unclear and passivated.

**Figure 7:**
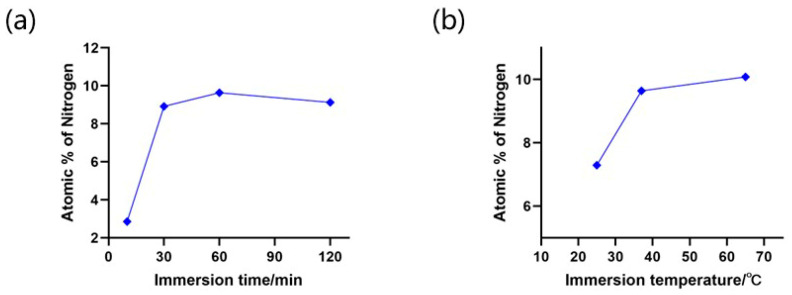
The atomic weights of nitrogen for the sandblasted microchip under (**a**) different BSA immersion times at 37 °C and (**b**) different BSA incubation temperatures for 60 min.

In addition, EDS analysis results indicated that the concentration of the absorbed BSA was increased with prolonged coating time, reaching the maximum of 9.64% for 60 min immersion (Figure 7a). We also discovered higher temperatures promoted the BSA coating, as shown in Figure 7b, because sufficient heat facilitates the interaction between BSA and the silicon surface, leading to enhanced adsorption rate. To avoid protein denaturation by excessive temperature, we decided not to continue raising the coating temperature [54]. Based on the above results, we considered 65 °C for 60 min as the optimal BSA coating condition, which was applied in further experiments. The EDS spectrums generated by different coating conditions are shown in Figure S5.

### 3.5. Comparison of COVID-19 PCR between DRIE and Sandblasted Microchips

**Figure 8:**
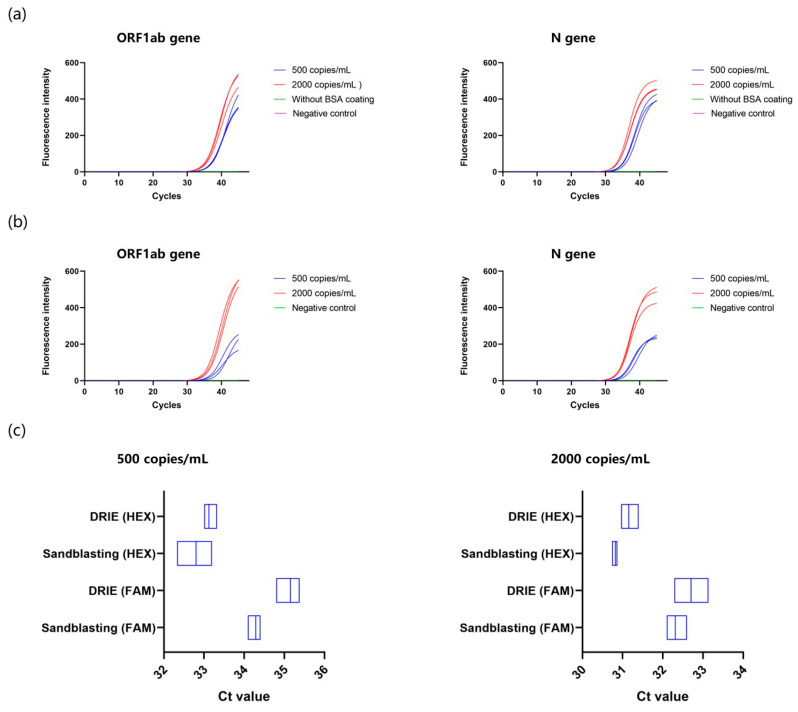
Amplification curves of ORF1ab gene and N gene of COVID-19 standard samples using (**a**) BSA-coated sandblasted and (**b**) DRIE microchips in the microdevice. (**c**) Comparison of the Ct values between two different fabrication methods of microchips. The reactions were conducted on the microdevice. Three replicates were set for each test. The BSA coating process of the sandblasted microchips took place at 65 °C for 60 min.

Using different concentrations of COVID-19 standard samples, we performed PCR tests to evaluate the biocompatibility of different microchips. As shown in Figure 8a,b, target genes were successfully amplified and detected on both silicon microchips. The results correctly revealed an inversely proportional relationship between Ct value and sample concentration. However, the sandblasted microchip without the BSA layer showed no amplification signal, since the rough surface nonspecifically absorbed the polymerase and nucleic acid molecules. We also found that sandblasted microchips exhibited about 60% higher fluorescence enhancement in 500 copies/mL samples with respect to DRIE silicon chips (Figure S4). This phenomenon indicates that BSA coating has the capability of mitigating the absorption effects caused by rough surfaces, even enhancing amplification efficiency. On the one hand, the saturated BSA layer serves as a protein adsorption competitor, which can lower the possibility of reactant adhesion on the sandblasted silicon. On the other hand, BSA can neutralize inhibiting substances in the PCR mixture, providing an amplification reaction with a stable and neutral environment. No amplification curve was detected for negative controls.

The box plots shown in Figure 8c displayed lower Ct values obtained on the sandblasted microchips compared with those generated by DRIE chips. With the same template and enzyme concentration, the earlier occurrence of the threshold means more productive reaction efficiency. It was noted that sandblasted microchips coated with BSA showed strong repeatability for medium and detection limit concentrations, demonstrating low chamber-to-chamber deviation in our microdevice. All Ct values are recorded in Table S2. In addition, the total reaction time of the COVID-19 test was around 20 min including reverse transcription, which is significantly shorter than conventional PCR analyzers [55]. Therefore, this sandblasted microchip-based microdevice is expected to offer a rapid, low-cost, sensitive, and reliable alternative for biomedical applications.

### 3.6. Multiplex PCR Validation on the Sandblasted Microchip

**Figure 9:**
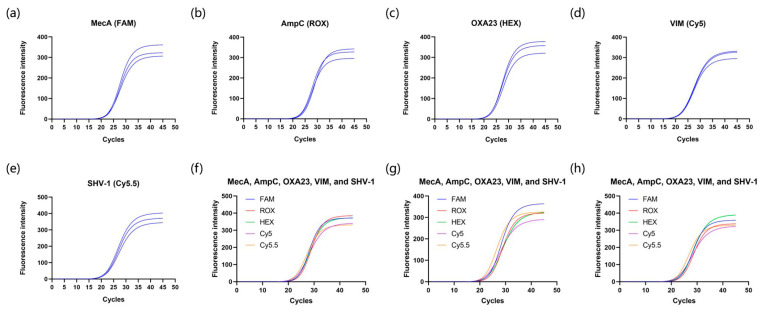
Amplification results of the multiplex PCR for screening resistance genes of endophthalmitis pathogen using single-gene controls, including (**a**) MecA, (**b**) AmpC, (**c**) OXA23, (**d**) VIM, and (**e**) SHV-1 genes and (**f**–**h**) multi-gene control (positive controls were provided in the kit). The assays were performed on our sandblasted microchip-based platform. Each sample was repeated three times in parallel. The sandblasted microchips mentioned above were coated with BSA at 65 °C for 60 min.

To further validate the mPCR feasibility of our sandblasting-based microdevices, we conducted multiple detections for drug-resistant genes of the endophthalmitis pathogen. In this experiment, we qualitatively detected single genes and multiple genes in reference samples. As illustrated in Figure 9, single and multiple targeted sequences displayed clear amplification signals, with high specificity and reproducibility. There was no mutual interference between different primer sets. For most of the results, the growth of fluorescence intensity ranged from 300 to 400 in the plateau phase, which indicated robust amplification efficiency of the microfabricated devices under different channels. The Ct values among the three replicates had a deviation of less than one for all resistance genes (Tables S3 and S4). There is no obvious difference in Ct values obtained from single-gene and multi-gene samples. Moreover, the detection time of mPCR was 50 min, which is longer than the previous PCR assay. This is mainly because enough time should be reserved for collecting the multiple signals during each cycle. Considering the high detection efficiency of mPCR, the duration time is acceptable and competitive among mainstream PCR devices [56].

## 4. Conclusions

In this work, we developed a sandblasted chip-based microdevice for point-of-care multiplex PCR diagnosis. The microheater and simplified optical module were employed to accomplish miniaturization. With a heating rate of 22.73 °C/s and uniform thermal homogeneity, the microdevice can complete a reverse transcription PCR within 20 min, showing superiority over commercial PCR instruments. Meanwhile, the portable optical system also exhibited reliable analysis capability in different fluorescence channels. These properties of our microdevice can provide crucial support for achieving on-site multiple PCR detection.

Moreover, another promising finding in our research is the cost-saving and easy-to-operate sandblasting technique for microchip fabrication. We formed microstructures with a hundreds-of-microns scale by an automatic sandblaster and hard photosensitive films. In comparison to conventional etching methods, the sandblasting process is capable of reducing manufacturing cost by 40~50%, presenting fewer environmental issues, and avoiding a lengthy process. However, the abrasive blasted surface showed a much rougher morphology than that etched by DRIE, resulting in unsatisfactory biocompatibility. Through the optimized BSA dip-coating, the coating layer can effectively facilitate nucleic acid amplification on rough microchips; the passivation effect remained stable after a standard PCR protocol. During the biocompatibility validation tests, sandblasted microchips displayed similar PCR efficiency to those of DRIE, even demonstrating enhanced amplification capabilities in detection limit concentration samples. Ultimately, multiple drug-resistant genes of endophthalmitis pathogens were specifically detected on the microdevice. Overall, our sandblasted microchip-based devices can prospectively advance a rapid, affordable, and dependable approach to multiplex molecular POCT and micromachining for biomedical microdevices.

## Figures and Tables

**Table 1:** Dimension information of the sandblasted microdevice and mainstream PCR analyzers.

Device	Size/mm	Weight/kg
Sandblasted microdevice	380 × 245 × 245	6.8
ABI 7500 fast	450 × 340 × 490	34
LightCycler^®^ 2.0 System	505 × 280 × 385	22

**Table 2:** Roughness data of microchips fabricated by sandblasting and DRIE using NanoScope Analysis 2.0 software.

Chip Type	Average Roughness (Ra)/nm	Root Mean Square Roughness (Rq)/nm	Image Rmax/nm
DRIE	2.41	3.04	25.4
Sandblasting	344	416	2211
Sandblasting (BSA coating)	196	256	1855
Sandblasting (BSA coating) after 45 thermal cycles	211	260	1527

**Table 3:** Cost comparison between the sandblasted and DRIE microchip fabrication processes (research use) [53].

No.	Sandblasted Microchip	Cost	DRIE Microchip	Cost
1	RapidMask handling	USD 10.8	Mask preparation	USD 128
2	Sandblasting	USD 50	Lithography	USD 145
3	Thermal oxidation	USD 145	DRIE	USD 145
4	Anodic bonding	USD 145	Thermal oxidation	USD 145
5	Dicing	USD 35	Anodic bonding	USD 145
6	-	-	Dicing	USD 35
Total (6-inch wafer)		USD 385.8		USD 743
Total cost per chip		USD 8.03		USD 15.48

## Data Availability

The data that support this study are available from the corresponding author upon reasonable request.

## Supplementary Materials

The following supporting information can be downloaded at: https://www.mdpi.com/article/10.3390/mi15080944/s1, Figure S1: Illustration of the plug-to-run sample loading and sealing process. (1, 3, and 5 are outlets; 2, 4, and 6 are inlets); Table S1: Fluorescence intensity of high, medium, and low concentrations of fluorescein under different channels (15 repetitions); Figure S2: Amplification curves of the fluorescence cross-talk test of (a) FAM, (b) HEX, (c) Cy5, (d) Cy5.5, and (e) ROX channels. The single-gene reactions using FAM, HEX, Cy5, Cy5.5, and ROX channels were set in well1, well2, well3, well4, and well5, respectively; Figure S3: (a) Photo of BSA coating stability test using 96-well heating block. The sandblasted silicon piece is highlighted in the red circle. (b) AFM scanning images of the BSA-coated sandblasted surface after stability test. The BSA coating was prepared at 65 °C for 60 min; Figure S4: Fluorescence increment of COVID-19 PCR tests (ORF1ab gene and N gene) on the sandblasted and DRIE microchips (average of the 3 repetitions). The sandblasted microchips were coated with BSA at 65 °C for 60 min; Figure S5: EDS spectrum images of different BSA dip-coating conditions. (a) 37 °C for 10 min; (b) 37 °C for 30 min; (c) 37 °C for 60 min; (d) 37 °C for 120 min; (e) 25 °C for 60 min; (f) 65 °C for 60 min; Table S2: Amplification results of different kinds of microchips using 500 copies/mL and 2000 copies/mL standard samples of COVID-19 (ORF1ab gene and N gene were labeled by FAM and HEX channels, respectively); Table S3: Ct values of the multiplex PCR assay on sandblasted microchips (single gene); Table S4: Ct values of the multiplex PCR assay on sandblasted microchips (multiple genes).

## References

[1] Hui D.S., Zumla A. (2019). Severe acute respiratory syndrome: Historical, epidemiologic, and clinical features. Infect. Dis. Clin..

[2] Goeijenbier M., Van Kampen J.J., Reusken C.B., Koopmans M.P., Van Gorp E.C. (2014). Ebola virus disease: A review on epidemiology, symptoms, treatment and pathogenesis. Neth. J. Med..

[3] Baloch S., Baloch M.A., Zheng T., Pei X. (2020). The coronavirus disease 2019 (COVID-19) pandemic. Tohoku J. Exp. Med..

[4] Dutta A., Banerjee N., Chaudhuri M., Chatterjee S. (2023). Nucleic Acid in Diagnostics. Nucleic Acid Biology and its Application in Human Diseases.

[5] Narasimhan V., Kim H., Lee S.H., Kang H., Siddique R.H., Park H., Wang Y.M., Choo H., Kim Y., Kumar S. (2023). Nucleic Acid Amplification-Based Technologies (NAAT)—Toward Accessible, Autonomous, and Mobile Diagnostics. Adv. Mater. Technol..

[6] Hawkins S.F., Guest P.C. (2017). Multiplex analyses using real-time quantitative PCR. Multiplex Biomarker Techniques: Methods and Applications.

[7] Clark T.W., Lindsley K., Wigmosta T.B., Bhagat A., Hemmert R.B., Uyei J., Timbrook T.T. (2023). Rapid multiplex PCR for respiratory viruses reduces time to result and improves clinical care: Results of a systematic review and meta-analysis. J. Infect..

[8] Ji K., Xu Y., Sun J., Huang M., Jia X., Jiang C., Feng Y. (2020). Harnessing efficient multiplex PCR methods to detect the expanding Tet (X) family of tigecycline resistance genes. Virulence.

[9] Zhu H., Zhang H., Xu Y., Laššáková S., Korabečná M., Neužil P. (2020). PCR past, present and future. Biotechniques.

[10] Akbari Kenari M., Rezvani Ghomi E., Akbari Kenari A., Arabi S.M.S., Deylami J., Ramakrishna S. (2022). Biomedical applications of microfluidic devices: Achievements and challenges. Polym. Adv. Technol..

[11] Auroux P.-A., Iossifidis D., Reyes D.R., Manz A. (2002). Micro total analysis systems. 2. Analytical standard operations and applications. Anal. Chem..

[12] Wang J., Jiang H., Pan L., Gu X., Xiao C., Liu P., Tang Y., Fang J., Li X., Lu C. (2023). Rapid on-site nucleic acid testing: On-chip sample preparation, amplification, and detection, and their integration into all-in-one systems. Front. Bioeng. Biotechnol..

[13] Wang X., Hong X.-Z., Li Y.-W., Li Y., Wang J., Chen P., Liu B.-F. (2022). Microfluidics-based strategies for molecular diagnostics of infectious diseases. Mil. Med. Res..

[14] Li Z., Xu X., Wang D., Jiang X. (2023). Recent advancements in nucleic acid detection with microfluidic chip for molecular diagnostics. TrAC Trends Anal. Chem..

[15] Gao D., Guo X., Yang Y., Shi H., Hao R., Wang S., Li Z.J., Zhao R., Song H. (2022). Microfluidic chip and isothermal amplification technologies for the detection of pathogenic nucleic acid. J. Biol. Eng..

[16] de Oliveira K.G., Estrela P.F.N., de Melo Mendes G., Dos Santos C.A., de Paula Silveira-Lacerda E., Duarte G.R.M. (2021). Rapid molecular diagnostics of COVID-19 by RT-LAMP in a centrifugal polystyrene-toner based microdevice with end-point visual detection. Analyst.

[17] Cojocaru R., Yaseen I., Unrau P.J., Lowe C.F., Ritchie G., Romney M.G., Sin D.D., Gill S., Slyadnev M. (2021). Microchip RT-PCR Detection of Nasopharyngeal SARS-CoV-2 Samples. J. Mol. Diagn..

[18] Rizzo M.G., Carnazza S., De Plano L.M., Franco D., Nicolò M.S., Zammuto V., Petralia S., Calabrese G., Gugliandolo C., Conoci S. (2021). Rapid detection of bacterial pathogens in blood through engineered phages-beads and integrated Real-Time PCR into MicroChip. Sens. Actuators B Chem..

[19] Manvi M., Swamy K.M. (2022). Microelectronic materials, microfabrication processes, micromechanical structural configuration based stiffness evaluation in MEMS: A review. Microelectron. Eng..

[20] Guo H., Cao S., Li L., Zhang X. (2022). A review on the mainstream through-silicon via etching methods. Mater. Sci. Semicond. Process..

[21] Juska V.B., Maxwell G., Estrela P., Pemble M.E., O’Riordan A. (2023). Silicon microfabrication technologies for biology integrated advance devices and interfaces. Biosens. Bioelectron..

[22] Damodara S., Shahriari S., Wu W.-I., Rezai P., Hsu H.-H., Selvaganapathy R., Li X., Zhou Y. (2021). 1—Materials and methods for microfabrication of microfluidic devices. Microfluidic Devices for Biomedical Applications.

[23] Deshmukh S.S., Goswami A. (2021). Recent developments in hot embossing–a review. Mater. Manuf. Process..

[24] Marks M.R., Cheong K.Y., Hassan Z. (2022). A review of laser ablation and dicing of Si wafers. Precis. Eng..

[25] Elvira K.S., Gielen F., Tsai S.S., Nightingale A.M. (2022). Materials and methods for droplet microfluidic device fabrication. Lab Chip.

[26] Zhou W., Dou M., Timilsina S.S., Xu F., Li X. (2021). Recent innovations in cost-effective polymer and paper hybrid microfluidic devices. Lab Chip.

[27] Ma J., Jiang L., Pan X., Ma H., Lin B., Qin J. (2010). A simple photolithography method for microfluidic device fabrication using sunlight as UV source. Microfluid. Nanofluidics.

[28] Martin A., Teychené S., Camy S., Aubin J. (2016). Fast and inexpensive method for the fabrication of transparent pressure-resistant microfluidic chips. Microfluid. Nanofluidics.

[29] Sreejith K.P., Sharma A.K., Basu P.K., Kottantharayil A. (2022). Etching methods for texturing industrial multi-crystalline silicon wafers: A comprehensive review. Sol. Energy Mater. Sol. Cells.

[30] Eun D.-S., Kong D.-Y., Kong S.H., Choi P., Shin J.-K., Lee J.-H. (2008). Fabrication of a based fluidic chip equipped with porous silicon filter and micro-channels. Jpn. J. Appl. Phys..

[31] Elias M., Dutoya A., Laborde A., Lecestre A., Montis C., Caselli L., Berti D., Lonetti B., Roux C., Joseph P. (2020). Microfluidic characterization of biomimetic membrane mechanics with an on-chip micropipette. Micro Nano Eng..

[32] Tong R., Zhang L.J., Song Q., Hu C.D., Chen X.E., Lou K., Gong X.Q., Gao Y.B., Wen W.J. (2019). A fully portable microchip real-time polymerase chain reaction for rapid detection of pathogen. Electrophoresis.

[33] Chen X.E., Song Q., Zhang B.N., Gao Y.B., Lou K., Liu Y.T., Wen W.J. (2021). A Rapid Digital PCR System with a Pressurized Thermal Cycler. Micromachines.

[34] Kodzius R., Xiao K., Wu J.B., Yi X., Gong X.Q., Foulds I.G., Wen W.J. (2012). Inhibitory effect of common microfluidic materials on PCR outcome. Sens. Actuators B-Chem..

[35] Christensen T.B., Pedersen C.M., Gröndahl K., Jensen T.G., Sekulovic A., Bang D.D., Wolff A. (2007). PCR biocompatibility of lab-on-a-chip and MEMS materials. J. Micromech. Microeng..

[36] Zhao J., Jiang E., Qi H., Ji S., Chen Z. (2020). A novel polishing method for single-crystal silicon using the cavitation rotary abrasive flow. Precis. Eng..

[37] Pal P., Swarnalatha V., Rao A.V.N., Pandey A.K., Tanaka H., Sato K. (2021). High speed silicon wet anisotropic etching for applications in bulk micromachining: A review. Micro Nano Syst. Lett..

[38] Vajpayee K., Dash H.R., Parekh P.B., Shukla R.K. (2023). PCR Inhibitors and Facilitators-Their Role in Forensic DNA Analysis. Forensic Sci. Int..

[39] Livak K.J., Schmittgen T.D. (2001). Analysis of relative gene expression data using real-time quantitative PCR and the 2^−ΔΔ*C*T^ method. Methods.

[40] Rutledge R.G. (2004). Sigmoidal curve-fitting redefines quantitative real-time PCR with the prospective of developing automated high-throughput applications. Nucleic Acids Res..

[41] Wu Z.-g., Zheng H.-y., Gu J., Li F., Lv R.-l., Deng Y.-y., Xu W.-z., Tong Y.-q. (2020). Effects of different temperature and time durations of virus inactivation on results of real-time fluorescence PCR testing of COVID-19 viruses. Curr. Med. Sci..

[42] Madadelahi M., Agarwal R., Martinez-Chapa S.O., Madou M.J. (2024). A roadmap to high-speed polymerase chain reaction (PCR): COVID-19 as a technology accelerator. Biosens. Bioelectron..

[43] Nguyen N.-T., Wereley S.T., Shaegh S.A.M. (2019). Fundamentals and Applications of Microfluidics.

[44] ABI. Applied Biosystems 7500 Fast and 7500 Real-Time PCR Systems. https://assets.thermofisher.com/TFS-Assets/LSG/brochures/cms_072493.pdf.

[45] Roche. LightCycler® 2.0 System. https://diagnostics.roche.com/global/en/products/instruments/lightcycler-2-0-ins-409.html#productSpecs.

[46] Labx. Price Information of the Commercial PCR Instruments. https://www.labx.com/product/applied-biosystems-7500.

[47] Jahed M., Gustavsson J.S., Larsson A. (2019). Precise setting of micro-cavity resonance wavelength by dry etching. J. Vac. Sci. Technol. B.

[48] Chen J.J., Qiu X.C. (2024). The effect of the surface passivation on polymerase chain reaction inside a continuous flow microfluidic chip. Microsyst. Technol..

[49] Marshall P.L., King J.L., Budowle B. (2015). Utility of amplification enhancers in low copy number DNA analysis. Int. J. Leg. Med..

[50] Liang Y., Wu J., Li Y., Li J., Ouyang Y., He Z., Zhao S. (2015). Enhancement of ginsenoside biosynthesis and secretion by Tween 80 in Panax ginseng hairy roots. Biotechnol. Appl. Biochem..

[51] Su Y., Chu H., Tian J., Du Z., Xu W. (2021). Insight into the nanomaterials enhancement mechanism of nucleic acid amplification reactions. TrAC Trends Anal. Chem..

[52] Karunanathie H., Kee P.S., Ng S.F., Kennedy M.A., Chua E.W. (2022). PCR enhancers: Types, mechanisms, and applications in long-range PCR. Biochimie.

[53] Nanosystem Fabrication Facility (CWB) H. The NFF (CWB) Laboratory Charging Scheme. https://nff.hkust.edu.hk/en/our-services/charging-scheme.html.

[54] Matsarskaia O., Bühl L., Beck C., Grimaldo M., Schweins R., Zhang F., Seydel T., Schreiber F., Roosen-Runge F. (2020). Evolution of the structure and dynamics of bovine serum albumin induced by thermal denaturation. Phys. Chem. Chem. Phys..

[55] Hossain M.W., Hossain M., Arafath K., Ety S.S., Shetu M.M.H., Kabir M., Noor F.A., Mannoor K. (2022). Real-Time fast PCR amplification using designated and conventional real time thermal cycler systems: COVID-19 perspective. PLoS ONE.

[56] Choi J.A., Bae S.M., Kim J.W., Lee K.J. (2020). Development of a Two Triplex Real-Time Polymerase Chain Reaction for Rapid Detection of Six Carbapenemase Genes in Enterobacteriaceae. Osong Public Health Res. Perspect..

