# Supplementary material for: Fabrication of Cost-Effective Microchip-Based Device Using Sandblasting Technique for Real-Time Multiplex PCR Detection
Source: Micromachines (Basel). 2024 Jul 24;15(8):944. doi: 10.3390/mi15080944 (PMC11356311; doi:10.3390/mi15080944)
Supplement: Supplementary file 1 [file micromachines-15-00944-s001.zip › micromachines-3095865-supplementary.pdf]

Fabrication of Cost-Effective Microchip-Based Device Using Sandblasting Technique for Real-Time Multiplex PCR Detection

Supplementary Materials

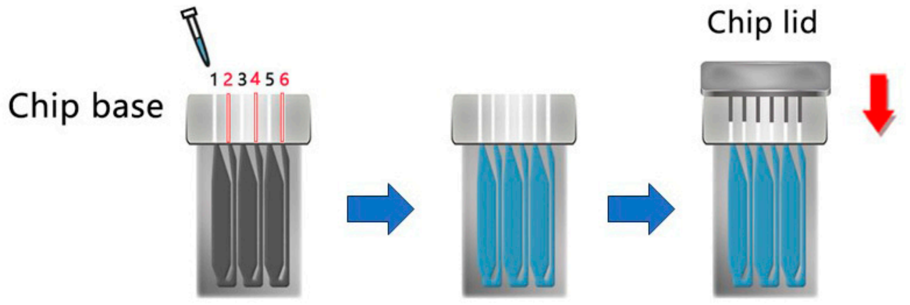

Figure S1. Illustration of the plug-to-run sample loading and sealing process. (1, 3, and 5 are outlets; 2, 4, and 6 are inlets).

Table S1. Fluorescence intensity of high, medium, and low concentrations of fluorescein under different channels (15 repetitions).

Fluorescence channel	Low Concentration	Medium Concentration	High Concentration
	26.072	47.699	117.060
	26.338	48.293	118.787
	26.028	47.991	118.863
	25.886	47.397	116.893
	25.550	46.759	115.485
	26.511	48.597	120.308
	26.448	48.792	120.489
FAM	25.146	46.167	114.272
	26.070	47.988	119.038
	25.063	45.814	113.377
	25.322	46.618	115.440
	25.662	46.874	115.681
	25.606	46.975	116.140
	25.489	46.539	114.641
	26.293	48.392	119.564
Average	25.832	47.393	117.069
Standard deviation	0.468	0.933	2.296
CV	1.811%	1.969%	1.961%
	32.468	120.750	248.330

	32.464	121.101	248.364
	32.547	121.237	248.386
	32.555	121.526	248.507
	32.304	120.412	248.181
	32.464	120.695	248.288
	32.369	120.340	248.168
HEX	32.485	121.195	248.362
	32.756	121.411	248.365
	32.358	120.438	248.177
	32.255	120.363	248.200
	32.236	120.147	248.166
	32.201	119.969	248.074
	32.429	121.080	248.415
	32.240	120.222	248.165
Average	32.409	120.726	248.277
Standard deviation	0.150	0.499	0.123
CV	0.464%	0.413%	0.050%
	10.989	16.568	162.338
	10.885	15.886	158.809
	10.846	16.135	160.391
	10.770	16.411	161.385
	10.736	16.324	161.235
	10.825	16.531	162.188
	10.742	16.366	161.114
ROX	10.773	15.814	158.405
	10.758	15.817	158.219
	10.729	16.264	160.946
	10.713	16.040	159.792
	10.742	16.209	160.585
	10.711	16.445	161.608
	10.713	16.290	160.839
	10.702	16.379	161.681
Average	10.776	16.232	160.636
Standard deviation	0.080	0.246	1.297
CV	0.739%	1.515%	0.808%
	4.780	34.225	180.722
	4.753	34.375	182.021
	4.760	34.369	182.298
	4.631	34.412	182.495
	4.837	34.680	182.979

Cy5	4.761	34.437	182.386
	4.802	34.753	183.489
	4.697	34.020	180.948
	4.722	34.379	182.955
	4.802	34.699	183.346
	4.786	34.563	183.003
	4.786	34.697	183.400
	4.828	34.810	183.815
	4.734	34.496	183.179
	4.808	34.621	183.896
Average	4.766	34.502	182.729
Standard deviation	0.054	0.216	0.941
CV	1.130%	0.627%	0.515%
Cy5.5	3.691	11.872	176.375
	3.810	12.193	180.618
	3.815	12.167	177.008
	3.803	12.075	174.095
	3.748	12.158	173.764
	3.767	12.030	171.009
	3.759	12.144	172.340
	3.819	12.193	176.660
	3.776	12.201	176.207
	3.735	12.027	171.057
	3.775	12.033	172.440
	3.787	11.962	170.007
	3.741	11.912	167.441
	3.747	11.919	169.038
	3.759	11.856	166.659
Average	3.769	12.050	172.981
Standard deviation	0.035	0.123	3.921
CV	0.926%	1.024%	2.267%

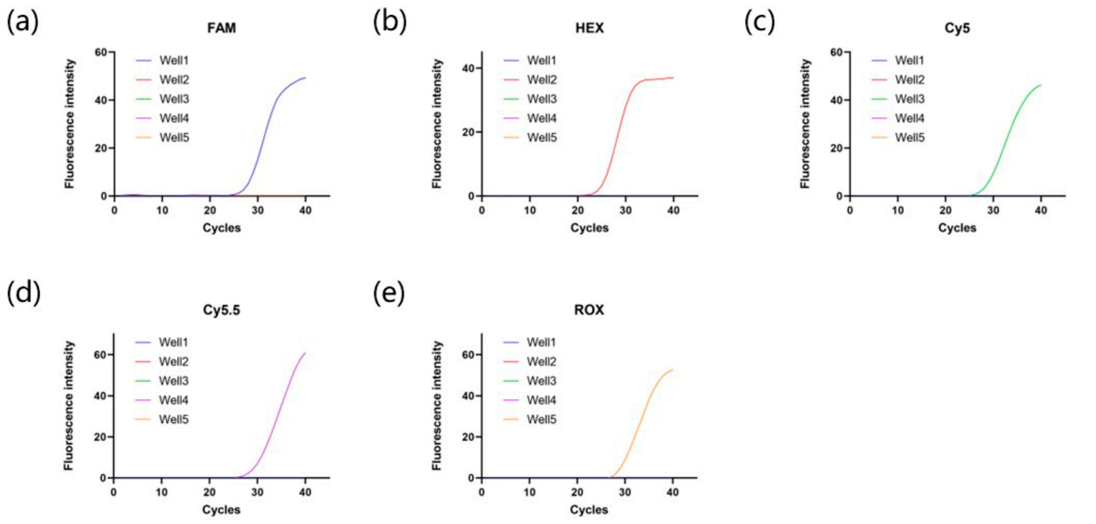

Figure S2. Amplification curves of the fluorescence cross-talk test of (a) FAM, (b) HEX, (c) Cy5, (d) Cy5.5, and (e) ROX channels. The single-gene reactions using FAM, HEX, Cy5, Cy5.5, and ROX channels were set in well1, well2, well3, well4, and well5, respectively.

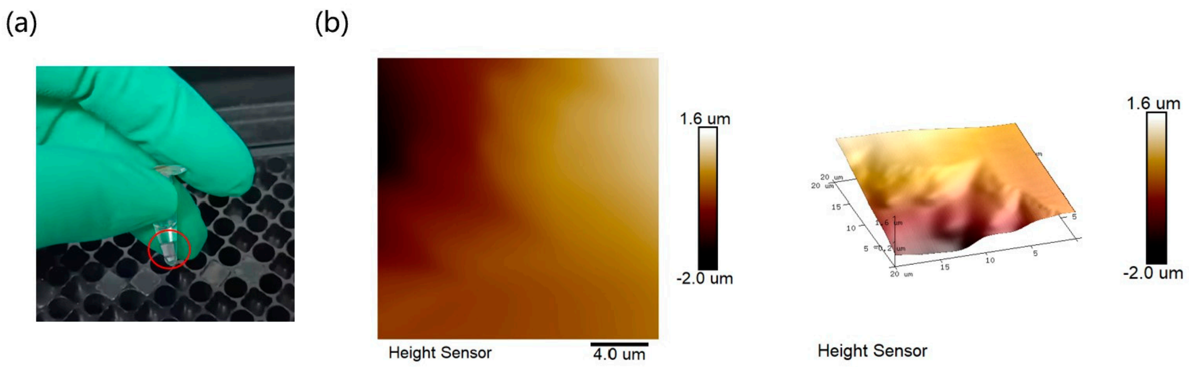

Figure S3. (a) Photo of BSA coating stability test using 96-well heating block. The sandblasted silicon piece is highlighted in the red circle. (b) AFM scanning images of the BSA-coated sandblasted surface after stability test. The BSA coating was prepared at 65 °C for 60 min.

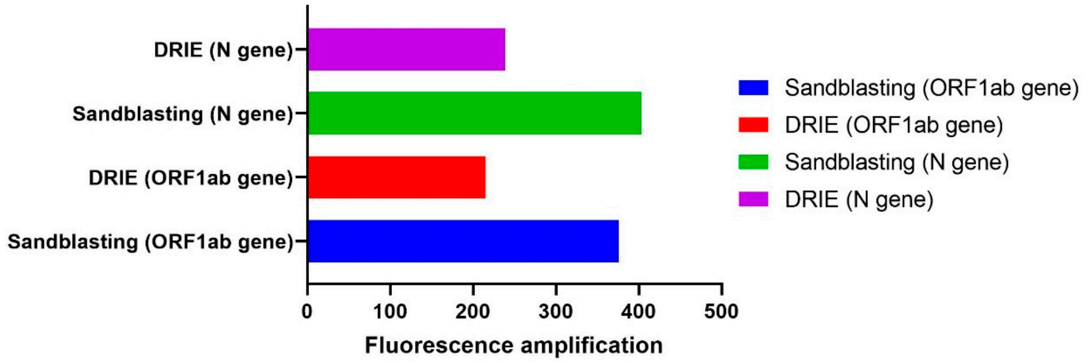

Figure S4. Fluorescence increment of COVID-19 PCR tests (ORF1ab gene and N gene) on the sandblasted and DRIE microchips (average of the 3 repetitions). The sandblasted microchips were coated with BSA at 65 °C for 60 min.

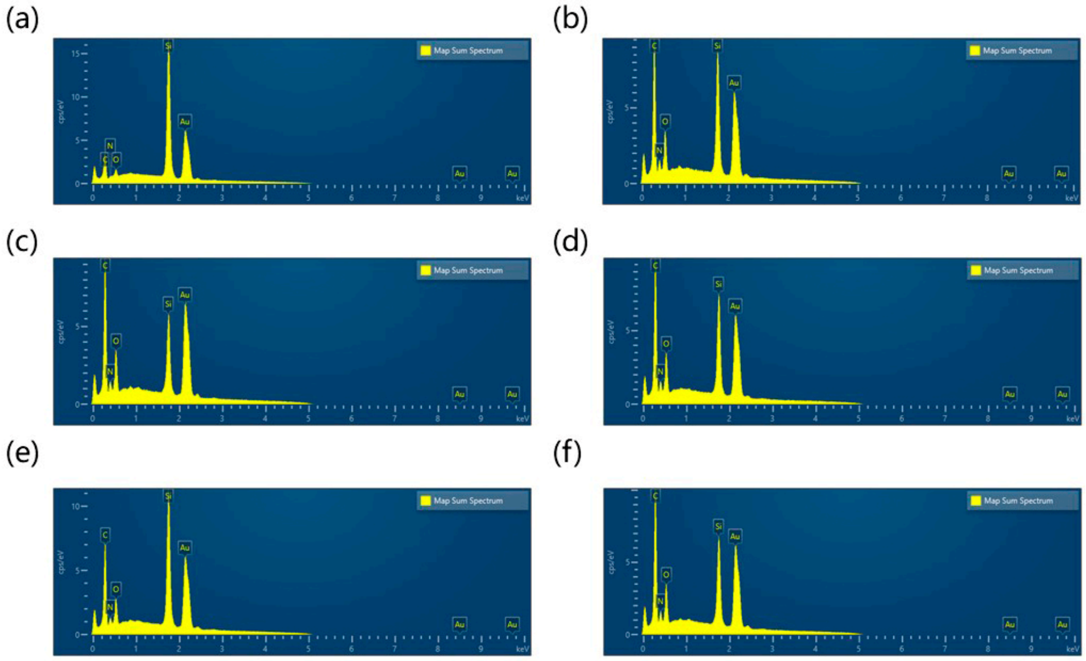

Figure S5. EDS spectrum images of different BSA dip-coating conditions. (a) 37 °C for 10 min; (b) 37 °C for 30 min; (c) 37 °C for 60 min; (d) 37 °C for 120 min; (e) 25 °C for 60 min; (f) 65 °C for 60 min.

Table S2 Amplification results of different kinds of microchips using 500 copies/mL and 2000 copies/mL standard samples of COVID-19 (ORF1ab gene and N gene were labeled by FAM and HEX channels, respectively).

No.	Chip type	Sample concentration	Ct (FAM)	Ct (HEX)
1	Sandblasting with BSA coating	500 copies/mL	34.38	33.2
2	Sandblasting with BSA coating	500 copies/mL	34.09	32.33
3	Sandblasting with BSA coating	500 copies/mL	34.41	32.87
4	Sandblasting with BSA coating	2000 copies/mL	32.1	30.87
5	Sandblasting with BSA coating	2000 copies/mL	32.23	30.74
6	Sandblasting with BSA coating	2000 copies/mL	32.6	30.86
7	Sandblasting with BSA coating	Negative control	NA	NA
8	Sandblasting	2000 copies/mL	NA	NA
9	DRIE	500 copies/mL	35.27	33.01
10	DRIE	500 copies/mL	34.8	33.33
11	DRIE	500 copies/mL	35.38	33.03
12	DRIE	2000 copies/mL	32.69	30.97
13	DRIE	2000 copies/mL	33.13	31.1
14	DRIE	2000 copies/mL	32.29	31.4
15	DRIE	Negative control	NA	NA

Table S3. Ct values of the multiplex PCR assay on sandblasted microchips (single gene).

No.	Components of positive sample	Fluorescence channel	Ct
1	MecA	FAM	20.66
2	MecA	FAM	21.08
3	MecA	FAM	20.71
4	AmpC	ROX	21.93
5	AmpC	ROX	21.71
6	AmpC	ROX	21.07
7	OXA23	HEX	20.86
8	OXA23	HEX	20.53
9	OXA23	HEX	20.07
10	VIM	Cy5	20.34
11	VIM	Cy5	20.17
12	VIM	Cy5	19.75
13	SHV-1	Cy5.5	19.31
14	SHV-1	Cy5.5	19.29
15	SHV-1	Cy5.5	18.85
16	Negative control	All channel	NA

Table S4. Ct values of the multiplex PCR assay on sandblasted microchips (multiple genes).

No.	Components of positive sample	Fluorescence channel	Ct
1	MecA, AmpC, OXA23, VIM, and SHV-1	FAM	21.58
		ROX	21.32
		HEX	20.63
		Cy5	20.45
		Cy5.5	19.66
2	MecA, AmpC, OXA23, VIM, and SHV-1	FAM	21.04
		ROX	20.89
		HEX	19.71
		Cy5	20.1
		Cy5.5	19.71
3	MecA, AmpC, OXA23, VIM, and SHV-1	FAM	21.24
		ROX	21.74
		HEX	20.19
		CY5	19.95
		Cy5.5	19.92
4	Negative control	All channel	NA
